# Flow virometry: recent advancements, best practices, and future frontiers

**DOI:** 10.1128/jvi.01717-24

**Published:** 2025-01-27

**Authors:** Claire Fernandes, Arvin T. Persaud, Deepa Chaphekhar, Jonathan Burnie, Carolyn Belanger, Vera A. Tang, Christina Guzzo

**Affiliations:** 1 Department of Biological Sciences, University of Toronto Scarborough 33530 , Toronto, Ontario, Canada; 2 Department of Cell and Systems Biology, University of Toronto 7938 , Toronto, Ontario, Canada; 3 Flow Cytometry and Virometry Core Facility, Department of Biochemistry, Microbiology and Immunology, Faculty of Medicine, University of Ottawa 151173 , Ottawa, Ontario, Canada; 4 Department of Immunology, Temerty Faculty of Medicine, University of Toronto 12366 , Toronto, Ontario, Canada; Universiteit Gent, Merelbeke, Belgium

**Keywords:** nanoparticles, virus-like particles, human immunodeficiency virus, flow virometry, nanoscale flow cytometry, calibrated flow cytometry, vaccine quality control, virus sorting, quantitative flow virometry, virus phenotyping, virion-incorporated proteins

## Abstract

The imperative for developing robust tools to detect, analyze, and characterize viruses has become increasingly evident as they continue to threaten human health. In this review, we focus on recent advancements in studying human viruses with flow virometry (FV), an emerging technique that has gained considerable momentum over the past 5 years. These advancements include the application of FV in viral surface phenotyping, viral protein functionality, virus sorting, vaccine development, and diagnostics. With examples illustrated using primary data from our recent studies, we demonstrate that FV is a powerful yet underutilized methodology that, when employed with best practices and experimental rigor, can be highly valuable for studying individual virion heterogeneity, virus phenotypes, and virus-antibody interactions. In this review, we also address the current challenges when performing FV studies, propose strategies to overcome these obstacles, and outline best practices for both new and experienced researchers. Finally, we discuss the promising future prospects of FV within the broader context of virology research.

## INTRODUCTION

Viruses are incredibly heterogeneous, exhibiting an extraordinary range of phenotypes that influence infection and disease (1, 2). Just one virus, among a myriad of diverse particles, can initiate infection, making the study of individual virion phenotypes crucial for understanding virus biology and developing effective therapies. Moreover, virus preparations are known to contain a multitude of diverse nanoparticles, including defective virions and extracellular vesicles. Despite this understanding, how these diverse nanoparticles contribute to viral function still remains largely elusive in virology today. In order to generate a deeper understanding of the unique roles that these diverse particles play in virus infection, it is critically important to visualize and quantify the heterogeneity within virus samples at the single-particle level.

Conventional virology techniques have typically relied on bulk particle analyses, which are only able to take the average statistic of a population of particles, and therefore cannot provide information on individual differences between nanoparticles within a given sample (3). Flow virometry (FV) is an underutilized tool that provides a powerful lens into individual virion heterogeneity by enabling visualization of all contributing particles to a given sample and the analysis of particular subpopulations of interest. Moreover, FV techniques include the capability to make multi-parametric analyses, which can reveal differences in surface protein phenotypes and protein conformations through targeted antibody staining, while also revealing inconsistencies in viral stock preparations that might be overlooked with classical bulk techniques. These unique advantages thus offer critical new insights into the heterogeneity of virus samples and subpopulations for targeted downstream applications.

In the past 5 years, interest and adoption of FV have surged. This is partly because of technological advancements that have helped overcome previous barriers in instrument sensitivity, as well as the emergence of new reagents specific to nanoscale flow cytometry. However, significant progress in this field has been driven by researchers optimizing existing cytometry instruments for small particle detection. Many of these early achievements and technological recommendations have been addressed in two earlier reviews by Lippé (4) and Zamora and Aguilar (5). Most recently, Tabler and Tilton (6) provided a concise overview of flow virometry best suited for those new to this emerging technique. Although not discussed in this article, many advances in FV are attributed to foundational studies analyzing virus particles in aquatic environmental samples (7–10), and much work is still being conducted in this realm today. This review herein focuses on the most recent advancements in FV over the past 5 years, including applications to study viral surface phenotypes, viral protein functionality, virus sorting, vaccines, and diagnostics. It also addresses ongoing challenges in FV assays, presents best practices for overcoming these challenges, and illustrates their utility with a comparative analysis of our own data from past studies. Finally, we discuss emerging opportunities to leverage FV for future discovery and scientific progress.

As an overarching theme, we hope to emphasize that critical consideration for further advancement in this field must have a focus on data calibration. Unlike with cells, where the cytometer settings are adjusted to fit the sample on scale, FV requires pushing the instrument settings to its limit of detection, allowing small particles like viruses to be detected wherever they inherently fall. Because individual instruments possess different thresholds of detection and acquisition settings, light scatter and fluorescence measurements of nanoparticles taken by different cytometers can vary significantly in their reported values. In an orchestra, each instrument must be precisely tuned to a standard frequency. Without this unified tuning, even the most beautiful melody can be rendered meaningless if it cannot be replicated or played with others. Just as a well-tuned instrument in an orchestra is essential for harmony in the ensemble, calibration in flow virometry ensures that data from different experiments and instruments can be reliably interpreted and applied. Therefore, this review aims to not only highlight the recent accomplishments in this emerging field but also to foster innovation by emphasizing the utility of calibration and best practices in FV experimentation, hopefully laying a tangible foundation for future discoveries and scientific advancement.

### A brief history on flow virometry

Initial experimentation using flow cytometers for virus detection began over 40 years ago using light scatter to discriminate between reoviruses, T2 bacteriophages, and latex spheres (11). Later, in the early 2000s, flow cytometric methods were also employed by marine biologists to enumerate viruses in aquatic samples (7–10). However, many of these early studies required customized cytometer configurations for virus detection or relied on fluorescent triggering due to the low light scatter sensitivity of instrumentation available (12, 13), which collectively limited the accessibility of flow virometry to the broader virology field at the time.

In 2013, Arakeylan et al. (14) used a commercially available cytometer to showcase the incorporation of host proteins into the HIV-1 envelope. Therein, they coined the term “flow virometry,” generating a high degree of interest for this technique, with similar approaches also applied to study the Dengue virus (15). Notably, the methodology employed by Arakeylan et al. (16) relied on a preliminary capture step that anchors viruses onto magnetic nanoparticles (MNPs) coated with antibodies targeting virus glycoproteins. This provided the advantage of enriching the detection of virus particles over other nanoparticle contaminants. However, this also introduced the possibility of impeding single particle detection (if more than one virus binds to a single MNP at a time), as well as potentially excluded the analyses of other virus populations not captured by the antibody-coupled MNPs. In later studies, direct and indirect labeling of virus particles with antibodies was undertaken for Nipah virus (17, 18), Junin virus (19), and cytomegalovirus (20), marking a large step forward in terms of new unbiased detection strategies. It should be noted that many of these studies relied on ultracentrifugation methods to purify virus populations, which may be problematic for some viruses, such as HIV-1 where it can induce viral glycoprotein shedding (21–23) and aggregate formation (24), both of which can potentially interfere with single particle analyses.

In 2016, Tang et al. (24) focused their efforts on the single particle analysis of vaccinia virus, showcasing FV as a means to differentiate single virions versus aggregates and highlighting this technique as a method to evaluate the physical attributes of a virus stock. Despite these advances, sensitivity with regard to surface protein detection on single virus particles still remains an inherent challenge, as viruses inherently possess a low number of surface antigens compared to cells, with many proteins present at or below the detection limit of many cytometers. Furthermore, even as instrumentation becomes more sensitive, current cytometers are still not able to detect the full breadth of nanoparticles within a single sample and have further limitations in accurately quantifying individual proteins present on a single particle. Moreover, variations in cytometer configurations (e.g., filter sets and laser wavelengths) and detection capabilities, differences in sample processing and/or labeling methods, combined with a lack of consensus for data reporting, make protein quantification on virus particles challenging. Concurrently, an extraordinary surge of research in the extracellular vesicle (EV) field has occurred (13, 25–27), with an emerging consensus among EV researchers that there is a need to establish guidelines for data rigor and reproducibility (28). It has become clear that controls and reporting standards are needed to ensure small-particle cytometry data can remain reliable and relatable over time and as instruments improve, providing impetus for the importance of data calibration.

In the context of this review, the term calibration refers to converting arbitrary units to standardized units using established reference materials. This process is critical to obtaining quantitative and reproducible measurements of virus count, size, and protein content with FV techniques and can be performed for both light scattering and fluorescence parameters. Light scatter calibration converts measurements of light scattering to estimates of particle diameter (nm) or scattering cross-sectional area (nm^2^) and uses beads in a range of sizes and well-defined material composition, which are acquired and calibrated with a software that uses Mie theory, the optical configuration of the cytometer, and the known refractive index of the virus (29–31). Fluorescence calibration is most commonly reported in units of Molecules of Equivalent Soluble Fluorophore (MESF) and requires beads with assigned fluorescence intensities that are stained with a known concentration of fluorophore, which should ideally match the antibody conjugate used for staining small particles (32). Briefly, these known fluorescence values are plotted against their corresponding arbitrary unit and a regression line is drawn through these points. The slope and *y*-intercept are used to convert the arbitrary values into a calibrated scale and assign each arbitrary unit a calibrated quantitative value (29, 32).

### Origins of quantitative flow virometry

Quantitative fluorescence measurements originated with CD4 quantification on T cells in HIV-1 infection (33–41), using QuantiBRITE phycoerythrin-labeled beads to quantify cellular surface antigen expression in units of Molecules of Equivalent Soluble Fluorophore. Although these methods have been established since the late 1990s, fluorescence calibration in flow cytometry applications remains relatively uncommon. Notably, two recent studies have harnessed fluorescence calibration to quantitatively map the expression of CD antigens on the surface of human immune cells (42, 43). These authors also used QuantiBRITE phycoerythrin-labeled beads to determine the number of PE molecules present. Although PE conjugation to mAbs typically maintains a 1:1 ratio of fluorophore to antibody (36, 41), the authors additionally tested for any deviations in this ratio using UltraComp eBeads Compensation Beads. These beads were used to capture each of the ~100 PE-conjugated mAbs and calculate a correction factor, which was then used to recalculate the antibody-binding capacity (ABC) for all 111 CD markers on all 47 defined cell subsets (43, 44). For small particles, fluorescence calibration has also been performed with QuantiBRITE PE MESF beads, enabling reporting of fluorescence intensity of surface antigen staining in standardized (i.e., calibrated) units (29, 45). Principles for fluorescence calibration have also been used to estimate the size of liposomes by Stoner et al. (46), in which fluorescence intensity from the membrane dye di-8-ANEPPS was used to approximate the surface area and diameter of small particles.

Light scatter calibration, which was first demonstrated by Fattacioli et al. (47) for small particles and was later applied to EVs by van der Pol et al. in 2012 (13), is used to convert arbitrary scattering intensities into approximations of particle size reported in calibrated units. However, the broader application of light scatter calibration in nanoscale flow cytometry was initially limited due to the complexity of normalizing light scatter signals using Mie Theory-based modeling. To simplify this process, a commercial light scatter calibration assay, Rosetta Calibration by Exometry, was introduced in 2015 (48). Rosetta Calibration uses Rosetta polystyrene beads for scatter calibration, which are purchased as a kit that includes software enabling fully automated light scatter calibration. This software provided a platform that can determine the light scatter-diameter relationship, specific to an individual flow cytometer (48). In 2019, FCMPass was introduced as a freely available software, which now enables calibration of both light scatter (30) and fluorescence (29). In contrast to Rosetta Calibration, FCMPass is semi-automated and allows one to use beads from any NIST size traceable standards of known diameter and refractive index (which can be purchased from a wide variety of manufacturers) and requires the user to determine and input the median scatter intensity of these beads (30). Later in 2020, the MIFlowCyt-EV position paper (49) was published*,* which has since been recommended as an important reporting framework in the most recent iterations of the Minimal Information for Studies of Extracellular Vesicles (50–52). The MIFlowCyt-EV report was the first to advocate for the simultaneous use of both fluorescence and light scatter calibration for nanoscale cytometry experimentation.

Following the best practices outlined in the MIFlowCyt-EV position paper (49), light scatter and fluorescence calibration techniques were introduced into the FV field by Welsh et al. (29). Welsh et al. (29) assayed a murine leukemia virus (MLV) construct containing a GFP-Env fusion protein, which was labeled with an anti-GFP-PE antibody. By simultaneously performing calibration for both fluorescence and light scatter, this study demonstrated the utility of calibration in generating highly reproducible quantification data from the same nanoparticle sample when collected on two distinct cytometry platforms. Detailed methods for running both fluorescence and light scatter calibration beads can be found in the methods section of Welsh et al.’s paper (29). Informed by this approach for our own investigations in HIV-1 biology, we applied this technique in the analysis of HIV-1 pseudoviruses (PVs) to provide quantitative estimates of human integrin α4β7, CD14, and PSGL-1 expression on the HIV-1 surface in standard units of PE MESF/nm^2^ (53). Maltseva and Langlois also described this technique and other recommendations for viral analyses with FV using MLV particles (54).

## RECENT ADVANCES AND APPLICATIONS OF FLOW VIROMETRY

### Phenotyping the viral surface

In initial studies, flow virometry showed promise for its utility in characterizing the surface antigen profiles of viruses (14, 55), and there have been many new studies demonstrating this application of FV over the past 5 years. Direct labeling techniques are most commonly applied in FV to detect human proteins present on viral surfaces (3, 19, 53–58–59), including engineered viral surface tags (59). The predominance of direct labeling techniques is supported by the abundance of commercially available fluorescence-conjugated antibodies that target these cell-specific antigens and/or tags. In 2020, our research group generated the first calibrated data set for quantitative analyses of HIV-incorporated surface proteins, with direct labeling of host proteins on HIV-1 virus particles (53). In 2021, Maltseva and Langlois used direct staining techniques in FV to reveal that host-derived tetraspanins, CD81 and CD63, as well as the lipid raft marker, Thy1.2, were incorporated on MLV particles containing a glycosylated Gag protein (58). In 2022, we used our optimized FV staining techniques and calibration protocols to further study virion-incorporated PSGL-1 in HIV-1 particles and validated our findings of PSGL-1 on virions from clinical samples *ex vivo* through complementary techniques (53, 57). Most recently, we demonstrated the potential for using calibrated FV in a discovery-driven approach, whereby we screened HIV virions for approximately 360 human antigens, using a commercially available flow cytometry-based cell staining kit on four different primary HIV-1 isolates (60). This work represented the first large-scale screening effort of viral surfaces with FV assays to date.

Indirect staining methods, where an unlabeled primary antibody is revealed via a secondary fluor-conjugated antibody, are less common, and these methods have largely been employed to target viral envelope glycoproteins (17, 20, 61–66), as fluor-conjugated primary antibodies for these antigens are not commonly commercially available. Most recently, our group employed indirect staining techniques to study the trimeric HIV-1 envelope glycoprotein (Env), using a panel of 85 unlabeled, monoclonal anti-Env antibodies targeting a diverse array of conformational epitopes, with differences detected in the presence/absence of soluble CD4 (67). Indirect staining methods in FV have also been used by others to target phosphatidylserine in an effort to interrogate the altered lipid profile of HIV-1 particles (61).

Accurately characterizing the presence of an antigen or specific epitope on the viral surface relies on the cytometer analyzing one single particle at a time. Therefore, meticulous attention should be applied to sample preparation, such as virus dilution and antibody titration, which are discussed in later sections of this review. Moreover, immunophenotyping is highly dependent on epitope accessibility, as well as antibody avidity and affinity. Therefore, fluorescence calibration is a crucial component as it allows for quantitative and consistent estimates of protein abundance and fluorescence intensity, enabling reliable comparisons across different instruments currently in use and over time as technology advances. For further details on these best practices, refer to “Best practices” herein.

### Assessing protein functionality

Flow virometry has been applied to study the functionality of proteins on viruses and can provide new knowledge of how these virus-associated proteins can influence virus biology. For example, our group recently used FV to demonstrate the ability of HIV-1 particles with incorporated CD14 to bind bioactive bacterial lipopolysaccharide (LPS), with evidence that LPS-loaded viruses can trigger inflammatory signaling in bystander immune cells (56). We also showed effective neutralization of LPS binding to HIV-1 particles upon pre-incubation of the virus with anti-CD14 antibodies before exposure to LPS (56). Collectively, this study demonstrated new applications for FV in assessing the ligand-binding capacity of viral surface proteins.

With respect to the functionality of the trimeric HIV-1 Env, FV has been previously employed to assess diverse conformations of Env (68), including differences in HIV-1 aggregates in comparison to their producer cells (62). Additionally, another recent study used flow cytometry approaches to detect antibody-mediated conformational changes in HIV-1 Env, which was achieved by detecting virus particles coupled to 1 µm fluorescent beads (69). Very recently, our investigations have demonstrated reliable sensitivity of FV techniques to detect induced changes in the HIV-1 Env trimer conformations on the surface of individual intact virions. These fluctuations were induced by incubating viruses in the presence or absence of soluble CD4 to trigger conformational changes in Env (67). In contrast to previous work in this area, we placed an emphasis on single virus analyses of particles in their native state by assessing viruses directly from culture supernatants to minimize experimental bias in Env evaluation (i.e., without coupling to magnetic or fluorescent beads, and no Env shedding from ultracentrifugation procedures). Therefore, this recent work presents a new application of quantitative FV, whereby we demonstrated the capacity to manipulate virus particles with the exogenous addition of a soluble virus receptor, and subsequently measured changes in spike conformations that can influence virus neutralization and infectivity (67).

FV has also been harnessed to study protease activity within HIV-1 particles. Bonar Michał et al. developed a FRET reporter, “Viral Protease Reporter”, to investigate the heterogeneity of protease activation in individual, patient-derived viruses using FV (70). They also used particle counts from FV to assess the impact of mutations on protease activity and viral particle release from infected cells. Importantly, using FV they were able to observe substantial inter-patient variability in protease activity among 36 unique patient-derived cloned isolates, which correlated with virus infectivity (70). The technique has since been used by this group to study the kinetics of protease activation in viral particle formation (71).

### Virus sorting

Past advances in flow virometry staining techniques coupled with technological advances in instrumentation have made it possible to sort virus particles (19, 55, 72, 73). Sorting homogenous virus populations can provide the advantage of recovering a viable virus sample that can be used for further downstream analyses of virus biology. For example, Lippé’s group (74, 75) has built upon their previously established sorting protocols exploring heterogeneity in herpes simplex virus (HSV) populations through staining with nucleic acid dyes to purify intracellular herpes virus intermediates (76), followed by downstream proteomic analyses to elucidate mechanistic differences of HSV capsid intermediates with different infectivity profiles (77). Furthermore, FV-based sorting methods have been applied by others to sort specific virus subpopulations via targeting distinct glycoproteins on the virus surface (55, 66), as well as to sort out virus aggregates with altered light scatter profiles (62, 78).

Sorting homogenous virus populations from heterogeneous samples remains a challenging endeavor with significant scope for improvement. Currently, there is a lack of established guidelines for data reporting, verification, and reproducibility in this emerging field, with only a handful of studies conducted in the past 5 years. Musich et al. (55) demonstrated purification of HIV particles from clinical samples and identified low post-sort yields as a key limitation during virus sorting protocols. Morales-Kastresana et al. (44, 45) presented promising preliminary work that further optimized virus sorting techniques, emphasizing the importance of systematically examining a “not gate” to exclude background reference noise from the sorting algorithm, which represents the scattered light arising from the laser:stream intercept in the absence of a particle. Therefore, this “not gate” signal encompasses electronic, fluidic, and optical noise generated by the cytometer. They posited that visualizing this background noise offers a partial representation of material below the detection range of the cytometer. This is important in virus-sorting protocols, as it helps assess whether aggregation or swarming occurs when virus samples are analyzed at high concentrations, minimizing the risk of coincident detection. Additionally, they advocated for the use of a high-sensitivity scatter parameter, rather than fluorescent triggering, which is commonly employed in previous virus-sorting studies using less sensitive cell sorters, as it provides a more comprehensive representation of the population, informing choices of sorting parameters and gating strategies (45). Moreover, herein, we also suggest that by employing light scatter calibration, one can enable estimates of scatter cross-section or diameter that can help reliably identify the population on different cytometers for post-sort acquisition and analyses.

Developing routine protocols in virus sorting for downstream applications to study virus biology involves key considerations, such as the time-consuming nature of sorting small particle subpopulations and the resulting reduction in virus concentration after sorting due to dilution in sheath fluid. Moreover, virus-labeling methods for gating and sorting can impact the infectivity of the recovered virus particles, as fluor-conjugated antibodies may interfere with virus binding to target receptors on cells. Despite these challenges, isolating a homogeneous viral subset is crucial for understanding how viral surface proteins impact virus infection, transmission, and pathology. Collectively, it is clear that virus sorting with FV techniques is an important area for future development, and it should be undertaken with proper controls, realistic expectations, and best practices in mind.

### Vaccine and therapeutic development

The recent SARS-CoV-2 pandemic has underscored the importance of adaptable and efficient manufacturing processes in vaccine development. A few prior studies have demonstrated the utility for FV to monitor virus particle production by detecting a high degree of heterogeneity in vaccine preparations (24) and providing information on particle concentrations (20). While at this time FV may not be suitable to entirely replace current monitoring strategies in vaccine provision, it has demonstrated a significant degree of utility by numerous vaccine studies over the past 5 years.

Here, we have summarized four key applications in this realm: (i) the ability to assess surface antigen incorporation, (ii) sorting target viral vectors from non-vector particles, (iii) physical titer measurements, and (iv) at-line process monitoring. First, the ability of FV to perform multi-parametric profiling analyses of surface antigens can be harnessed to assess protein incorporation and/or abundance on viral vaccine vectors (63, 65), with promising preliminary work done to simultaneously observe the incorporation of viral glycoproteins from Nipah and Hendra viruses on individual particles (64). Surface profiling by FV can also be useful in evaluating post-translational modifications. For example, a 2022 study used FV to validate the successful embedding of a polymer shield onto the surface of vaccinia virus particles to demonstrate their improved evasion of neutralizing antibodies (79). Second, in enabling users to visualize distinct particle subpopulations, FV techniques can be used to sort specific virus subpopulations of interest from bulk preparations, such as isolating particles containing proteins derived from recombinant vectors from particles devoid of these proteins (80). A third distinct application of FV in vaccine studies is the ability to count all intact virus particles in a given virus preparation. Traditional assays used to measure viral titers rely on bulk analytical methods, such as ELISA or RT-PCR, which cannot provide information on single intact virus particles or virus aggregation (24). TCID50 or plaque assays only indicate viral particles that can infect and/or lyse host cells (81). Since non-infectious particles can also influence host immune responses, Renner et al. (81) demonstrated that measuring total MLV particle counts by FV offers a quick and simple way to gain a more fulsome understanding of all particles present within a sample and enables a correlation of particle counts with infectious units. A similar technique was used in a study employing retrovirus particles to deliver Cas-9 single guide RNA and the CAR gene in CAR-T cell tumor immunotherapy (82). Interestingly, a recent 2021 study by Niu et al. (83) demonstrated that physical virus titers of adenovirus measured by FV are more closely correlated with the infectious units determined by plaque assay rather than viral genome titers as quantified by qPCR. Finally, FV can also be applied as an additional monitoring step for the purposes of vaccine quality control in large-scale commercial manufacturing (65). For example, in 2021, Ricci et al. (84) successfully applied FV to identify damaged particles, in the live-attenuated and recombinant virus vaccine, ERVEBO. Herein, they demonstrated the precise temperature control required to maintain particle integrity using the altered scatter and fluorescent profiles of particles subjected to various temperatures to detect damaged particles with FV (84).

Importantly, Yi et al. (85) emphasized that establishing FV as a tool to inform on various parameters in vaccine provision requires extensive reference data under various manufacturing conditions, including employing orthogonal techniques in parallel. Then, relying on the assumption that consistent processes maintain constant particle counts, fluorescence, and light scatter, any significant changes in these parameters could be used to signal whether a deviation in particle quality control or an introduction of contaminants has occurred during manufacturing. Moreover, in this context, fluorescence and light scatter calibration should be of utmost importance for future use of FV in vaccine provision to allow for inter-instrument comparisons. Thus, employing fluorescence and light scatter calibration complements the stringent quality standards that are crucial for the development of effective and reliable vaccines and therapeutics.

### Diagnostics

Accurate and early disease diagnosis is essential for effective therapeutic treatment and control of viral infections. FV holds promise as a supplementary methodology for high-throughput viral detection and has recently been proposed as an additional tool for immediate and sensitive detection of virus particles in biological samples by some preliminary studies. Razafimahefa et al. have recently advocated for using flow cytometry as a tool for human norovirus screening in biological fluid and food samples (86). This study employed antibody-coated magnetic beads to capture human norovirus from patient stool samples and artificially infected *Mytilus edulis* samples (86, 87). Similarly, flow cytometry approaches were also applied to detect bluetongue viruses in cattle serum, utilizing a bulk capture method, with antibody-coated 7.5 µm microspheres (88). It should be noted here that both these studies do not analyze single virus particles. Rather, they employ antibody-mediated capture using large beads as a preliminary enrichment step. This method is advantageous for diagnostic applications as it permits purification by specifically capturing virus particles and removing traces of debris from complex matrices. However, it relies on the availability of high-affinity antibodies that must sufficiently capture enough particles for detection.

Another important consideration for scaling up the use of FV to detect viruses in biological samples is the accessibility to specialized cytometry instruments. Currently, many commercially available instruments that enable sensitive single nanoparticle analyses are large, relatively expensive, and often require operators with special expertise in nanoscale flow cytometry, which, taken together, can limit its accessibility in certain diagnostic settings. However, one recent report has demonstrated the use of a custom-built “flow virometry reader” to detect SARS-CoV-2 from the saliva of infected individuals, displaying the potential for becoming a fast and portable diagnostic tool in low-resource settings or point-of-care locations (89).

Importantly, when using FV to detect viruses directly from various biological fluids for diagnostic purposes, the properties of those fluids must also be taken into consideration. No published work to date has compared how the composition of different bodily fluids alters virus staining in FV applications. For example, viruses in low-viscosity saliva samples may be more accessible to antibody-based staining compared to viruses in high-viscosity fluids like breast milk or genital secretions. Therefore, special attention to concentration measures, sample processing, and acquisition parameters is critical to control before embarking on large-scale investigations in clinical samples.

## CURRENT CHALLENGES

### Low abundance antigen detection on virus particles

**Fig 1:**
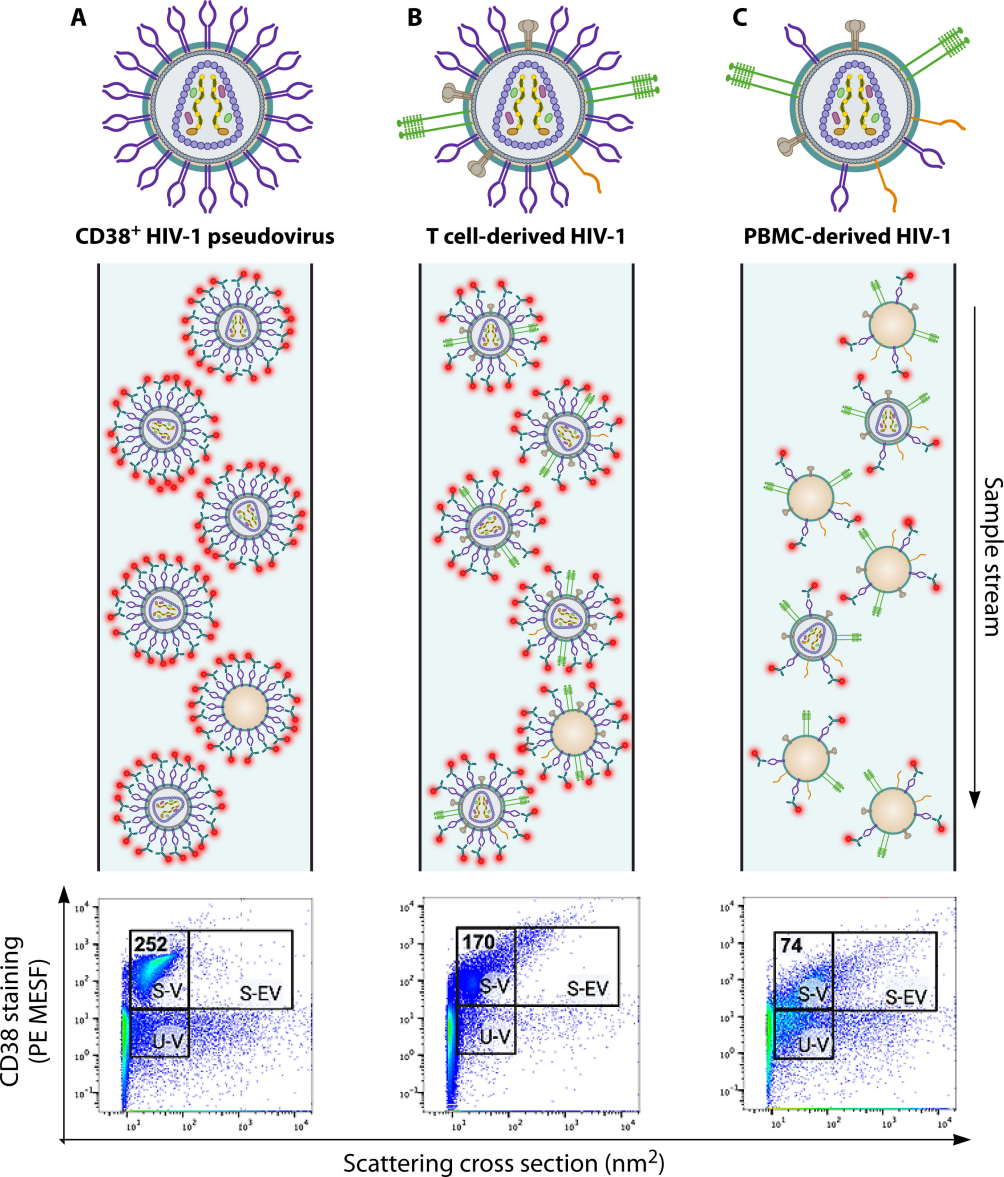
Surface antigen detection on different virus models with flow virometry. (**A**) CD38+ HIV-1 pseudoviruses produced by transfection of HEK293T cells are depicted as a single virus particle with high levels of surface CD38 (top) and as a sample stream running through the cytometer (middle). The representative pseudocolor dot plot (bottom) displays a tight, monodisperse, stained virus population in the upper left PE-positive gate. Fluorescence data (*y*-axis) were calibrated to produce quantitative units of PE MESF, which estimates the number of PE fluorophores present and, indirectly, indicates the number of antigens detected on a single viral particle. Side scatter data (*x*-axis) were calibrated to produce units of scattering cross-section, which relates to the size and refractive index of virus particles. Data are displayed in pseudocolor dot plots to allow for the visualization of virus and vesicle populations. The lower gate denotes unstained virus (**U-V**). The left and right upper gates display stained virus (**S-V**) and stained extracellular vesicles (**S-EV**), respectively. (**B**) HIV viruses produced via infection of the PM1 CD4+ T cell line, depicted as a single virus particle with endogenous levels of surface CD38 and other T cell antigens (top), with a heterogenous virus sample stream shown, including more contaminating EVs than what was observed in panel A. The representative pseudocolor dot plots display a disperse stained virus population, containing a wide range of events that appear outside of the virus staining gate, reprinting more particle heterogeneity. (**C**) HIV viruses produced via infection of primary peripheral blood mononuclear cells, depicted as a single virus particle with lower levels of all surface proteins (top) and a representative sample stream showing the highest numbers of EVs. Here, the representative pseudocolor dot plot displays a highly heterogeneous array of events, and the stained virus population shows a lower shift in fluorescence above background fluorescent noise levels. All samples displayed on dot plots were acquired for 1 minute at a flow rate of 10 µL/min. The dense population of events to the left of the gates on all plots represents the instrument’s optical noise. Particles depicted as empty shells (absence of internal viral capsids) represent defective virus particles and/or extracellular vesicles.

Different methods of virus culture and production can significantly impact the number of surface antigens appearing on viral surfaces and the heterogeneity of particles in a given virus sample. Thus, the selection of virus model systems should be a key consideration when performing immunophenotyping studies (67). Moreover, factors such as serum concentration, cell density, cycling, and activation state can impact the expression of surface antigens in cell culture, thus affecting the surface antigen profile acquired by enveloped virions. Additionally, additives such as phenol red may contribute to background fluorescence in some cytometer channels if not diluted or removed. To illustrate this, Fig. 1 compares different virus production systems for preparing HIV-1 viruses and virus-like particles, with each approach yielding visibly different levels of viral surface proteins, particle counts, and particle heterogeneity.

Figure 1A depicts an HIV-1 pseudovirus sample, created through co-transfection of HEK293T producer cells with an HIV-1 expression plasmid and a plasmid encoding a human protein of interest, CD38. While HEK293T cells do not endogenously express CD38, this protein is naturally expressed on a variety of leukocytes, including CD4+ T cells (90). Therefore, this pseudovirus production method generates a virus model where CD38 is overexpressed relative to their endogenous levels on CD4+ T cells. Consequently, the stained pseudovirus populations typically shift far above background fluorescence levels, with nearly all events appearing in the upper left positive stained virus (S-V) gate (Fig. 1A). In contrast, Fig. 1B and C display comparative staining for CD38 displayed on HIV-1 particles produced via natural infection of a CD4^+^ T cell line or primary peripheral blood mononuclear cells (PBMCs), respectively. These viruses incorporate lower levels of CD38, in comparison to the overexpression model in Fig. 1A, as a reflection of the lower levels of endogenous CD38 expression on the surface of these cell types (60). This is also mirrored quantitatively in the dot plots, whereby the calibrated units of fluorescence (molecules of equivalent soluble fluorophore) associated with virus particles were highest for the pseudoviruses (252 MESF) and lower for the viruses produced in CD4^+^T cell lines (170 MESF) and PBMC (74 MESF), whereby the PE MESF values provide an estimate of the number of protein molecules present on individual virus particles. Furthermore, many PBMC-derived virus particles (Fig. 1C), which contain the lowest number of CD38 antigens, do not effectively shift the virus particles entirely above the background fluorescence levels, with many particles remaining in the lower left gate (denoted as unstained virus, U-V) that overlaps with background fluorescence. This is in line with our previous observations, wherein viruses propagated in primary cells commonly contain ~10–20 protein molecules of interest per individual virus particle and are difficult to discern from fluorescent background signals, as they overlap with instrument noise (57, 60).

### Contributions of extracellular vesicles in virus preparations

Extracellular vesicles are small lipid-enclosed structures released by various cell types and display vast heterogeneity in size, membrane composition, surface proteins, and internal cargo. However, many of these properties overlap with those of viruses, especially during the course of infection (91), making it difficult to distinguish viruses from EVs. These heterogeneic EVs and viruses exist in a continuum within viral stock preparations (91–96), and their influence on FV data has long been a challenge for researchers (4, 5). While some groups have developed methods to enhance the detection of viruses over EVs (78, 97), their close resemblance in size and antigenic profile makes it challenging to confidently exclude all EVs from virus samples.

The contributing presence of EVs in virus data acquisition is not a unique challenge to FV. However, unlike conventional tools, FV enables users to visualize all particles present within a sample, which can reveal aspects of sample heterogeneity and complexity that are not typically observed or addressed in bulk analyses. As exemplified in Fig. 1B and C, the difference in staining and scatter profiles of a virus produced in a CD4^+^ T cell line versus PBMCs is visually distinct, with a diverse scattering cross-sectional spread that can be seen across the dot plots. Therefore, the unique capability of FV to visualize all particles within a given sample can be advantageous to investigators by providing important insight into the composition and heterogeneity of a virus sample.

Continuing to develop the tools, including reagents and protocols, to discriminate EVs from viruses in FV analyses will allow us to further tease apart the unique biological role that each of these particles play. For example, sucrose density gradients have been previously used to separate HSV-1 and HCoV-OC43 from EVs (98, 99). However, it might also be simultaneously important to consider that even when the optimal purification steps are employed, a virus preparation may never be entirely devoid of contaminating EVs. Therefore, at the current time, it is imperative to mitigate and acknowledge the contributions of EVs to the analyses of virus particles. This also includes performing the appropriate orthogonal techniques (e.g., immunocapture assays, RT-qPCR, ELISA, Western blot, etc.) to complement data acquired with FV according to one’s scientific question(100). Moreover, it is also critical to include the appropriate EV controls, such as staining performed on matched, mock-infected cell culture supernatants.

### Lack of commercially available reference materials for small particles

Reference materials, in the form of well-characterized calibration materials, are critically important for data reliability and reproducibility, as they support the conversion of arbitrary units to standardized units through data calibration. However, standard reference materials for multiple parameters, including physical (size and refractive index) and biochemical (surface proteins and internal cargo) features still need to be developed for viruses and EVs specifically, so that they match the respective inherent properties of small biological particles (44, 101). Indeed, it has been well documented that polystyrene and silica beads that are commonly used as sizing reference materials are very different in scattering properties from more flexible biological particles like EVs and viruses (53, 59, 73). In early attempts to address these challenges, Geeurickx et al. created a virus-based construct, which share similarities such as size, buoyant density, and refractive indices to that of EVs (99). Thus, this was proposed to be used as a biological reference material in efforts to standardize small-particle measurements for nanoscale flow cytometry (102, 103). In parallel, many other studies in the EV field have demonstrated the utility of similar virus-based particles (29, 59, 104, 105) or other nanoparticles as reference materials (106–108).

**Fig 2:**
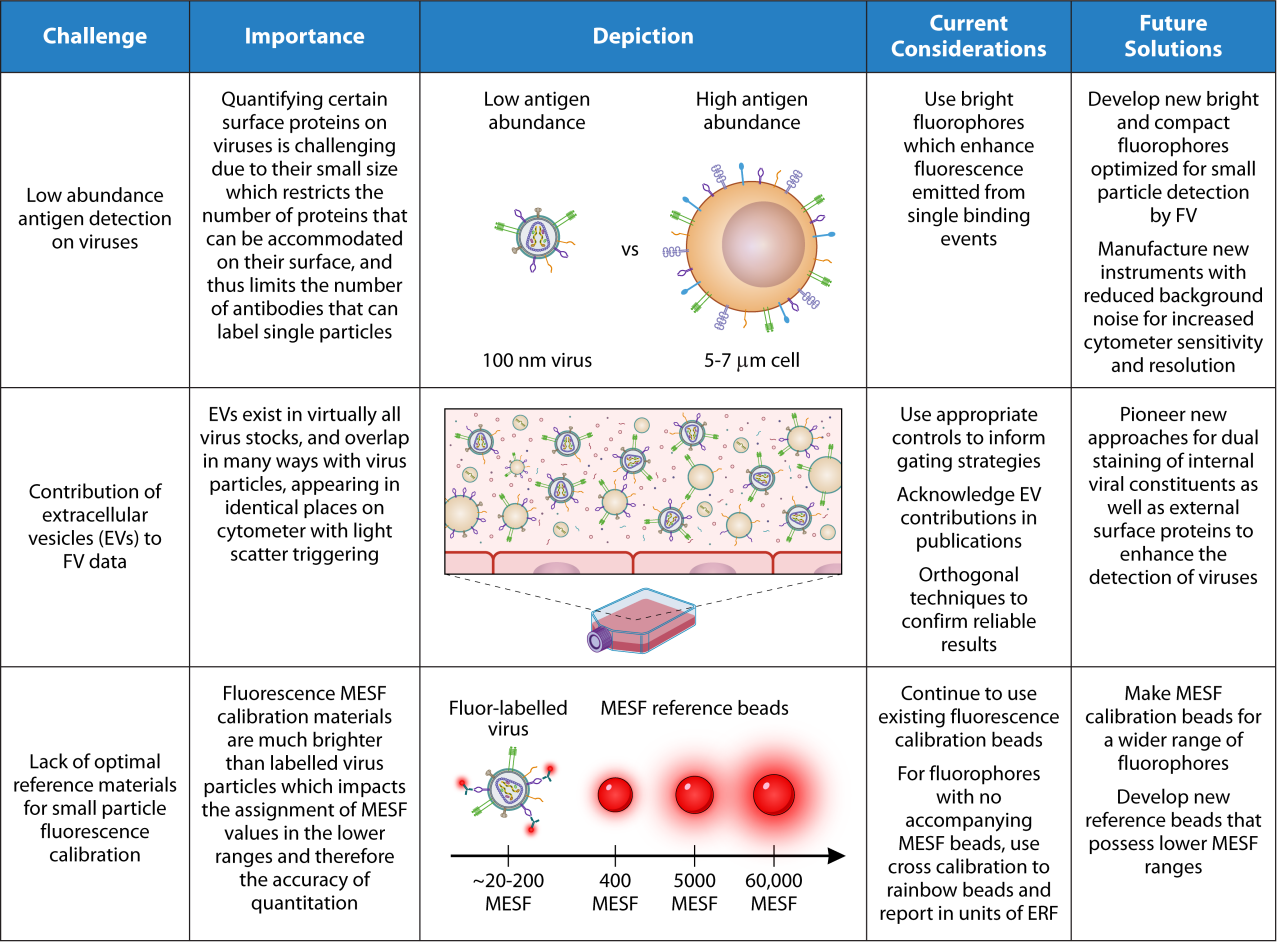
Current challenges in flow virometry.

Notably, many of the reference materials commonly used for fluorescence calibration in FV were initially developed for use in cells and typically possess much higher MESF values than possible for nanoparticles (Fig. 2). Because these values are extrapolated for application to a much dimmer small particle population, this can introduce errors that may affect the accuracy of MESF assignment in the lower ranges of fluorescence detection, which was recently demonstrated in a study on EVs (109). It is also important to note that MESF values reported for stained particle samples are only as accurate as the MESF values assigned to the reference beads, which can vary between manufacturers and lots. Therefore, it should be recognized that this approach for fluorescence calibration provides an estimation of true value, though it may not reflect the exact value. Although this leaves current assignments susceptible to minor inaccuracies, by reporting calibrated units alongside reference material specifications (e.g., catalog and lot number) and MESF standards, this will allow for future cross-calibration achievements across different types of fluorescence reference materials. Therefore, fluorescence calibration is important to establish a standardized method that enables researchers to obtain reproducible quantified data sets, thereby enabling more controlled comparisons across studies within the broader research community, which can collectively fuel collaboration and research advancements.

Additionally, a large majority of fluorophores lack matched quantitative fluorescence reference materials, which are fluorescent bead populations labeled with known amounts of this fluorophore, enabling the assignment of measured fluorescent intensity in standardized units of MESF (44). It is also important to note that larger fluorophores, specifically PE, typically have a fluor:protein (F/P) ratio of 1:1 due to steric hindrance and low conjugation efficiencies (29, 36, 41, 42, 53). However, this ratio does not apply for all conjugates, and the impact of fluor choice should be a major consideration in assay development in small-particle flow cytometry. This is echoed by a recent publication that found that the F/P ratio significantly influenced the detection of tissue factor on extracellular vesicles isolated from mesenchymal stem cells or plasma isolated from COVID-19-infected patients (110). Moreover, for applications that necessitate extremely precise levels of quantitation, the antibodies employed should be assayed by spectrophotometry to validate the fluor/protein ratio (44). Furthermore, since antibodies used in our studies are bivalent, the quantitation with appropriate MESF calibration materials may vary by a factor of two (36). Currently, it is recommended that for conjugates with no corresponding reference materials, multi-peak rainbow beads can be used to calibrate into units of equivalent reference fluorophore (32, 44). However, a more quantitative alternative fluorescence calibration method for immunofluorescence labeling is using antibody bead capture. This method uses beads bearing calibrated numbers of immunoglobulin binding molecules. These beads are first stained with a conjugated antibody of interest and then analyzed under the same conditions as samples stained with the same fluor-conjugated antibody. Using a similar method to MESF assignment, which employs linear regression, arbitrary fluorescence intensities can then be related to the number of antibody capture sites on the beads, and thus the number of antibodies per particle, in units of ABC (44). Despite this, there is still a need for manufacturers to co-develop new generations of calibration materials to support fluorescence data calibration and improve quantitative FV analyses for nanoscale particles.

## BEST PRACTICES

### Optimizing single virion detection: acquisition and sample preparation

To ensure optimal performance in routine viral detection, it is imperative to strictly adhere to the manufacturers’ cytometer cleaning protocols. Indeed, many nanoscale flow users will argue that additional cleaning is needed before running their samples (97). For example, our group will typically run buffer for 5–10 minutes, at different flow rates, to reduce the event rate to its lowest achievable levels before sample acquisition for the day. This may be especially necessary if residual cellular or tissue debris remains from previous experiments, which can potentially compromise virus detection. Next, if trying to determine whether your cytometer can indeed detect your small particles of interest and optimize performance, there are discrete and tested protocols that have been outlined in a recent “small particle optimization pipeline” (32). This pipeline outlines a systematic process to determine the optimal light scatter detector settings, threshold, limit of detection, and fluorescence detector settings that are specific for your cytometer and sample of interest. A workflow for optimizing one’s cytometer for detecting small particles can be found in Fig. 1 of Cook et al*.*’s manuscript (32).

**Fig 3:**
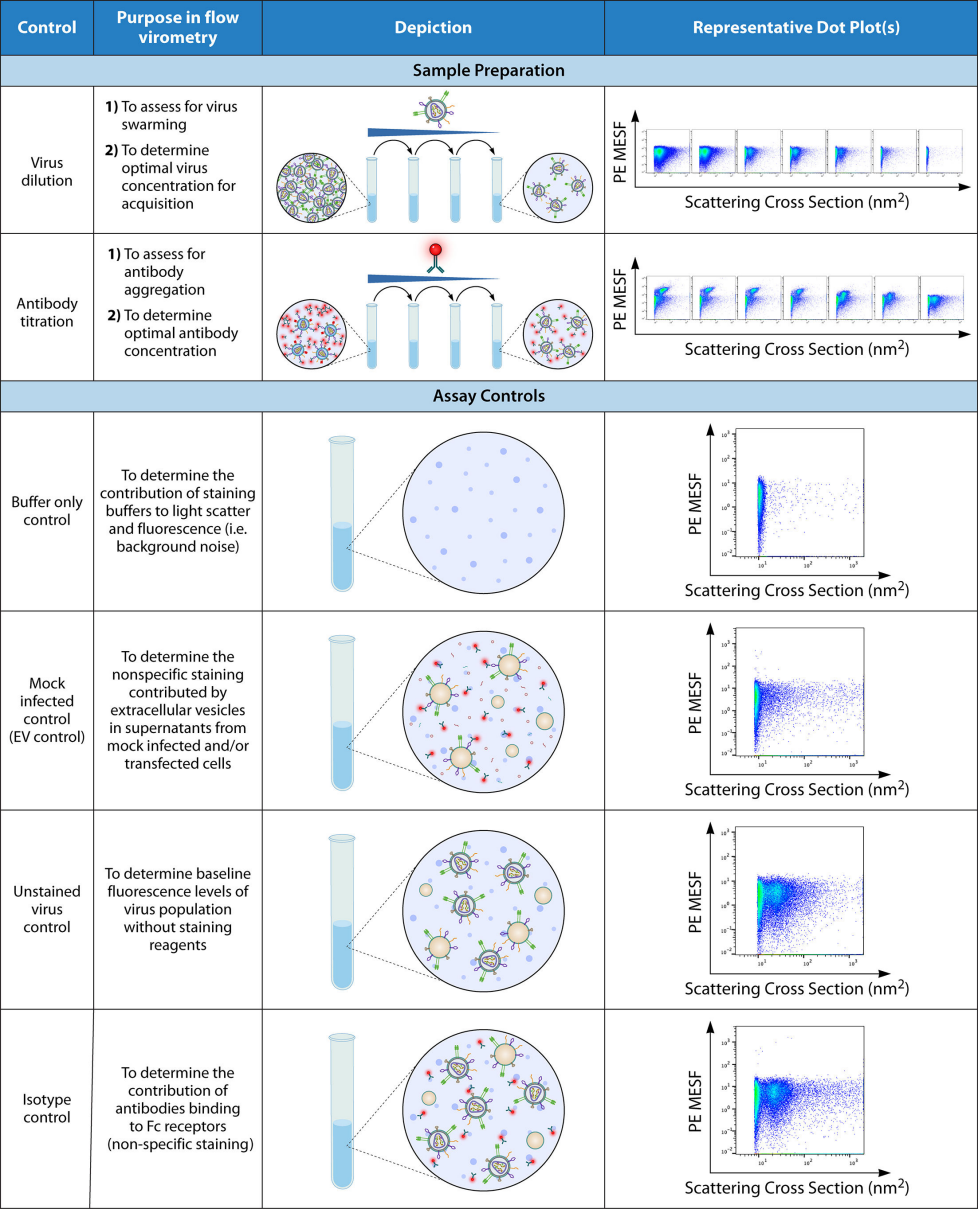
Best practices and controls for flow virometry experimentation.

Once the cytometer is ready for sample acquisition, an important consideration is the possibility of coincidence (111) or more recently coined “swarming” in nanoparticle analyses (13). When numerous small particles pass simultaneously through the interrogation point of the cytometer at once, this incorrectly generates a single event signal, increasing the electronic abort rate and ultimately underestimates the measured particle concentration and compromising single-particle analyses (13, 97). Therefore, especially concentrated virus samples must be diluted appropriately and always run at the slowest acquisition rate possible to reduce these occurrences. Second, it is also best practice to titrate all antibodies used for staining. This is particularly important for small particle staining protocols in which wash steps are not entirely necessary since viruses of nanoscale sizes are too small to be effectively pelleted on a bench-top centrifuge. Using excessive amounts of antibody, coincidental detection of unbound antibody aggregates, as well as a high level of background fluorescence, which collectively can mask dim antigen detection and reduce the detection of positive virus staining. These best practices and recommended assay controls are summarized in Fig. 3. It is important to note that viral analyses via flow virometry generally does not require sample purification and/or concentration, and wash steps are often omitted in small particle flow cytometry (29, 53, 56, 57, 60, 67). However, procedures such as ultracentrifugation, density gradient centrifugation, size-exclusion chromatography, and ultrafiltration may be needed in some cases to remove unbound reagents, such as anticoagulants, as well as reagents added prior to staining (such as fixatives). For a discussion on these procedures, Welsh et al.’s compendium cites many studies in which these techniques were used on extracellular vesicles (44). When employing any additional purification methods, careful consideration should be taken to ensure that a significant amount of particles are not lost during these procedures and that single-particle analysis is maintained.

### Achieving accurate and reliable reporting: importance of sample controls and data calibration

The recent MIFlowCyt-EV position paper outlines essential metadata, controls, calibration, and data reporting standards for small particle measurements of EVs using flow cytometry (49). Herein, Fig. 3 summarizes a range of controls recommended for the specific purpose of virus analysis. Importantly, we additionally recommend that a mock-infected (or mock-transfected) cell culture supernatant control is employed as one additional indicator for the contributions of EVs to virus preparations (54). For further information, a recent and comprehensive review by Welsh et al. also describes the utilization of these controls, as well as contains vital information on a broad range of attributes pertinent to nanoscale flow cytometry theory and application, including recommendations for small particle sample preparations (44). This valuable reference is applicable for researchers both new and experienced in FV techniques. Additionally, when publishing small particle cytometry data sets, all data should also be linked to a publicly available online flow repository (e.g., FlowRepository.org or Genboree.org/nano-ui/, etc.) and reported with a corresponding reference number for data transparency and accessibility. Furthermore, it is best practice to report flow data with the MIFlowCyt-EV checklist appended in publications to ensure optimal transparency and experimental information for others to reproduce and interpret the study data (49). A workflow for the experimental design of a flow cytometry experiment for small particles can be found in Fig. 10 of Welsh et al*.*’s compendium (44).

**Fig 4:**
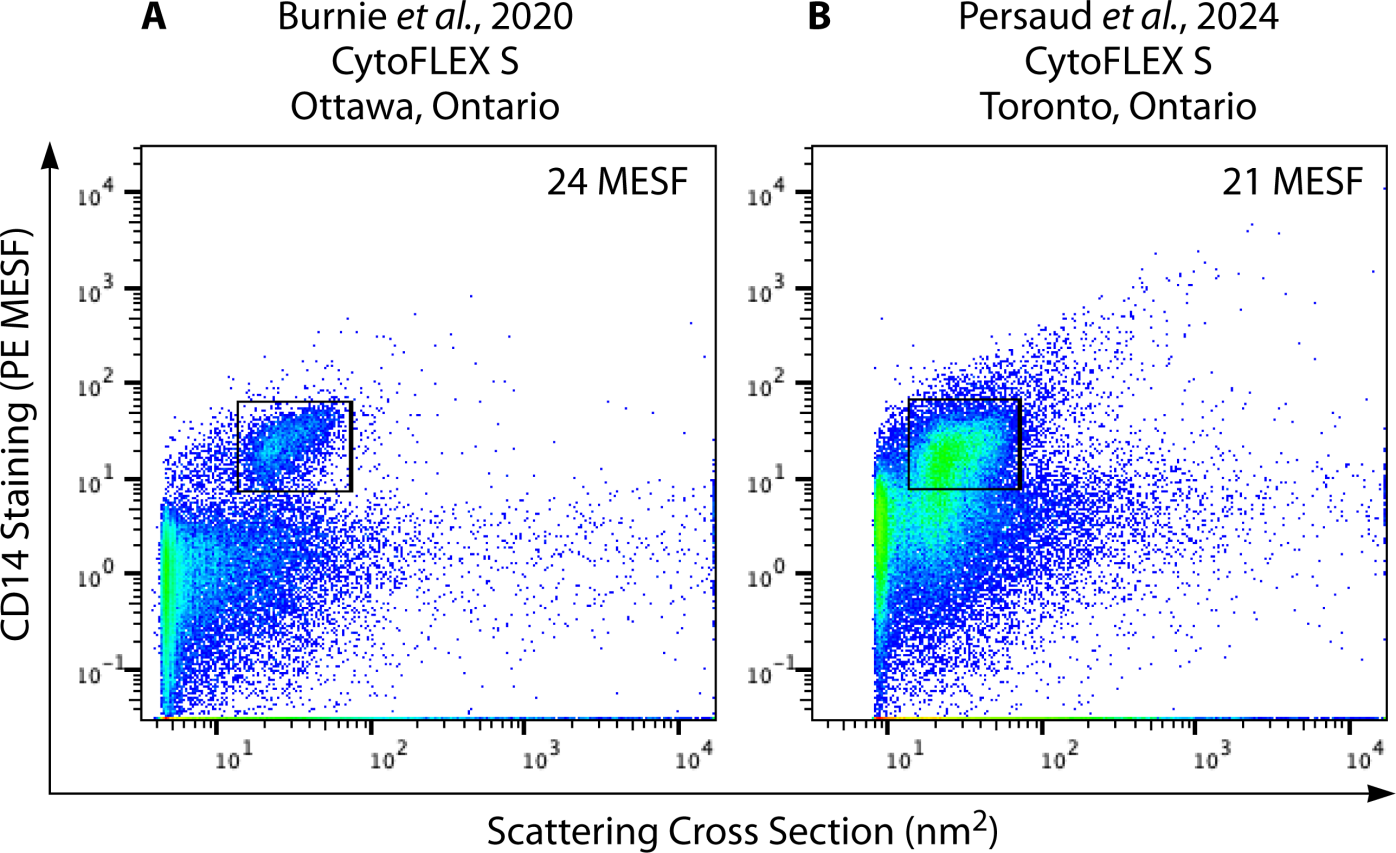
Calibration supports reproducible CD14 quantification on two different HIV-1 pseudovirus stocks, even when experiments were performed 4 years apart. Black gates indicate positive staining for CD14 on HIV-1 pseudovirus populations. PE MESF values are enumerated on each plot, describing the mean MESF detected in the stained virus gate. (**A**) These data were acquired by Burnie et al. in 2020 (53), with raw data retrieved from Fig. 4 within this prior manuscript. (**B**) These data were acquired by Persaud et al. in 2024 (56), with raw data retrieved from Fig. S1A (0.25 µg condition). Both data sets were re-analyzed here to present them in parallel with identical gates and their respective mean MESF values. Gate coordinates (***X*,*Y***) are as follows: top left (14,57), top right (78, 57), bottom left (14, 7), and bottom right (78, 7).

To illustrate the reliability and reproducibility of calibration efforts, Fig. 4 presents two similar FV data sets generated by our group in two separate publications, over 4 years apart. Here, Fig. 4A and 4B display pseudocolor dot plots of HIV-1 pseudoviruses engineered to display surface CD14 (CD14 + PV) stained with the same PE-conjugated anti-CD14 antibody. Both CD14 + PVs were produced with identical transfection protocols employed by different lab members. In 2020, Burnie et al. (53) first reported an estimate of ~20 molecules (20 ± 12.1 MESF; Fig. 4A) of CD14 on each PV particle, and more recently, Persaud et al. (56) reported an estimate of ~22 molecules of CD14 per virion (22 ± 9.91 MESF; Fig. 4B). Herein, we have re-analyzed the raw data from each publication to display graphs with identical axes and gating strategies, which have marginally altered their MESF values from their original published values. The values are now reported as 24 MESF and 21 MESF in Fig. 4A and 4B, respectively.

Notably, these two studies were performed by two different researchers, with two distinct preparations of viral stocks stored as frozen aliquots, with different viral titers, and on two different instruments; Fig. 4A was acquired in Ottawa, Ontario on a Beckman Coulter CytoFLEX S, and Fig. 4B was acquired on an identical instrument model, located in Toronto, Ontario. Remarkably, despite variations in scatter thresholds, detector settings, configuration, and acquisition settings between cytometers, the calibrated PE MESF values remain highly concordant. Furthermore, the stained virus populations are located in near identical positions on the two dot plots, despite differences in background fluorescence levels and particle heterogeneity. This exemplifies the utility of calibrating FV data sets, showing how calibration allows users to report quantitative measurements with a high level of reproducibility, despite various operational variables among experiments.

## FUTURE FRONTIERS

Flow virometry is an exciting tool that holds promising potential for diverse applications across virology fields. Moreover, with recent advances in cytometry instrumentation, this technique can be performed with many flow cytometers that are already present at most major core facilities. As discussed here, FV has yielded many advances in a wide range of applications, including viral protein phenotyping and functional analysis, vaccine production, diagnostics, and virus sorting. Here, we aimed to raise awareness that by using the appropriate controls and data calibration, FV experimentation produces reliable, reproducible, and statistically robust results. It is our hope that going forward, this technique can be added as a standard tool in virology studies. Moreover, as this methodology becomes more prevalent and refined, it could drive manufacturing advancements in instrumentation, reagents, and reference materials that are better optimized for detecting small biological particles like viruses and EVs. These innovations will enhance the capabilities and utility of FV, which can enable this technique to aid in addressing longstanding questions in virology. An important example of this potential lies in using FV to understand viral heterogeneity.

Many defective viruses are present in virus stocks (112–114), and these particles can still encode viral proteins despite lacking a full-length genome, and many of these particles can remain structurally intact (112). This suggests that functional and defective particles can be structurally similar and, therefore, share similar scatter profiles, despite having defective intravirion components. While cell permeabilization protocols for intracellular staining via flow cytometry are routine, this field is largely unexplored with respect to virion analyses by FV. Although some significant work has been done in this area within the EV field, with super-resolution microscopy techniques (115), optimization would still be required to develop virus-specific protocols for FV that permit permeabilization of virus particles, while maintaining surface protein antigenicity and virus particle integrity. These protocols would ideally allow for specific staining of internal virus components as well as external surface epitopes. This may be a challenging endeavor, simply due to the size of antibodies, their available fluorophore conjugates, and the limited space inside viral particles. Therefore, in developing these new experimental approaches, it may also be useful to consider using fragmented antibodies or single-chain variable fragments to more easily gain access to the virus interior (44).

Novel methods in multi-color immunophenotyping may also generate a more comprehensive understanding of the profile of surface antigens on enveloped viruses, which acquire a portion of the host cell plasma membrane upon budding from infected cells and can, therefore, acquire an antigenic phenotype that reflects that of the infected cell. In doing so, careful consideration of antibody staining competition, physical restraints in size or available target molecules/epitopes, as well as necessary compensation controls must be performed. Few groups have attempted to directly stain multiple antigens on single virions at once (19, 55, 59, 64), and further developments are necessary before this strategy could be routinely employed for staining viruses in clinical samples from infected patients as a future frontier in therapeutic monitoring. For example, in the EV field, multi-color labeling strategies have been employed by one recent study to immunophenotype and identify distinct extracellular vesicle subpopulations in porcine seminal plasma (116). In the short term, multi-color immunophenotyping of viruses with FV could offer a novel, non-invasive technique to determine which tissues or cell types *in vivo* are most likely the key source and/or reservoir of circulating viruses. This could be particularly useful in studying viral dynamics in people living with HIV who have undergone analytical treatment interruption, where a rebound of viral infection from the latent reservoir typically occurs after treatment cessation. Since it is challenging to identify a single antigenic marker of a distinct cell type, simultaneously targeting multiple cellular antigens as well as viral antigens can perhaps provide enhanced insight into the tissue source of where virions originate from, which can aid in identifying persistent viral reservoirs that are a crucial target in HIV cure strategies.

In conclusion, flow virometry holds the promise of yielding not only the laudable achievements outlined herein but also an enduring potential as a powerful new approach for significant advancements and discoveries in virology research.
